# An Innovative Cloud-Fog-Based Smart Grid Scheme for Efficient Resource Utilization

**DOI:** 10.3390/s23041752

**Published:** 2023-02-04

**Authors:** Fahad Alsokhiry, Andres Annuk, Mohamed A. Mohamed, Manoel Marinho

**Affiliations:** 1 Department of Electrical and Computer Engineering, Faculty of Engineering, King Abdulaziz University, Jeddah 21589, Saudi Arabia; 2 K. A. CARE Energy Research and Innovation Center, King Abdulaziz University, Jeddah 21589, Saudi Arabia; 3 Institute of Forestry and Engineering, Estonian University of Life Sciences, 51006 Tartu, Estonia; 4 Electrical Engineering Department, Faculty of Engineering, Minia University, Minia 61519, Egypt; 5 Polytechnic School of Pernambuco, University of Pernambuco (UPE), Recife 50720-001, PE, Brazil

**Keywords:** cloud computing, fog computing, improved differential evolution, gray wolf optimization, efficient resource utilization, smart grid

## Abstract

Smart grids (SGs) enhance the effectiveness, reliability, resilience, and energy-efficient operation of electrical networks. Nonetheless, SGs suffer from big data transactions which limit their capabilities and can cause delays in the optimal operation and management tasks. Therefore, it is clear that a fast and reliable architecture is needed to make big data management in SGs more efficient. This paper assesses the optimal operation of the SGs using cloud computing (CC), fog computing, and resource allocation to enhance the management problem. Technically, big data management makes SG more efficient if cloud and fog computing (CFC) are integrated. The integration of fog computing (FC) with CC minimizes cloud burden and maximizes resource allocation. There are three key features for the proposed fog layer: awareness of position, short latency, and mobility. Moreover, a CFC-driven framework is proposed to manage data among different agents. In order to make the system more efficient, FC allocates virtual machines (VMs) according to load-balancing techniques. In addition, the present study proposes a hybrid gray wolf differential evolution optimization algorithm (HGWDE) that brings gray wolf optimization (GWO) and improved differential evolution (IDE) together. Simulation results conducted in MATLAB verify the efficiency of the suggested algorithm according to the high data transaction and computational time. According to the results, the response time of HGWDE is 54 ms, 82.1 ms, and 81.6 ms faster than particle swarm optimization (PSO), differential evolution (DE), and GWO. HGWDE’s processing time is 53 ms, 81.2 ms, and 80.6 ms faster than PSO, DE, and GWO. Although GWO is a bit more efficient than HGWDE, the difference is not very significant.

## 1. Introduction

Smart grids (SG) are essential components of smart communities. A fast and efficient communication system to monitor and control power consumption is used in these grids to establish a smart and distributed system that can control power needs economically and sustainably [1]. In this regard, the Internet of Energy (IoE) could be thought of as a combination of SG and the Internet of Things (IoT) [2]. IoE aims to simplify the power trade among users by providing an effective structure. Internet controls and observes intermittent and distributed power production and storage logically. A wide variety of loads and sources will be allowed to trade power through the IoE, such as distributed energy storage, renewable energy resources (RERs), as well as residential and industrial users. Cloud computing (CC) is required to process, access, store, and manage robust processing sources. Therefore, IoT and CC can be grouped together to form IoE platforms capable of empowering pervasive sensing. By storing and utilizing sensing data coherently, intelligent observation and robust handling of the detection of data streams can be accomplished. With more smart appliances, latency and response time in CC increase, leading to deviations for several delay-sensitive devices and smart appliances [3]. In [4], authors developed realistic measures for assessing the physical structure of the cloud-fog-edge layers based on the reliability approach. They used the fault tree and Markov chain model to provide quantitative solutions. Ref. [5] suggested fog computing (FC) to address such problems. CC is dragged to the edge or corner of the network by fog or edge computing. By preprocessing the data, delay restrictions can be achieved, when meeting the scalability, interoperability, consistency, and enhanced system connectivity. Awareness of position, minimal delay, geographical distribution, a large number of appliances, mobility, real-time devices, and heterogeneity are some of the best attributes of FC [6]. The smart device system faces several issues, such as delayed demands, source-restricted devices, and network bandwidth. As the appliances have been linked to the internet, some safety issues arise, such as keeping source-restricted appliances safe, ensuring security, updating smart appliances, evaluating the safety state of large distributed systems accurately, and responding to vulnerabilities without any insurmountable problems which cannot be met by CC. Ref. [7] depicted the FC as a networking structure dispensing computing, storage, control, and system management near the appliances of consumers. The purpose of the study is to present a feasible framework for SG by integrating two new technologies: cloud and fog computing (CFC). Based on the proposed idea, a CFC-driven SG scheme was discussed in the paper, along with load-balancing strategies for handling SG consumer requirements.

SG structure is observable when two-way communication network systems are combined with electrical grids [8]. SGs are capable of providing electricity to consumers gradually and efficiently with the help of information and communication technology. According to SG technology, there are three main parts within the system, namely distribution, transmission, and production [9]. Obsolete power plants make up the production side. In addition, electric power must be distributed to consumers by the transmission feeders. In order to reduce environmental issues and the number of transmission losses caused by power plants, green energy or RERs have been introduced and gained popularity in recent years. Energy usage is monitored by the most important features of SG utilizing a variety of optimization strategies for the consumer [10,11]. Assuring the price, peak-to-average ratio, and consumer satisfaction are met by scheduling devices with various consumption profiles. Further, demand response management is emerging as an important empowerment technology for SGs.

In the literature, there are numerous studies that support the CC paradigm, such as ref. [12]. Ref. [13] compared CC with traditional approaches, recognizing several technical and nontechnical challenges and CC alternatives. The CC has been progressing quickly, but it has faced several problems, including long delays, poor reliability, mobility, awareness of position, and privacy issues [14,15]. Ref. [6] offered FC as a solution to the issues mentioned above. FC offers excellent solutions regarding latency, processing time, reliability, and so on. Technically, congestion and overburdening are caused by too many requests. This arbitrariness has its biggest drawback in task allocation. As a result of the random task allocation, there will be a few overloaded and a few underloaded processors [16]. As a result of load balancing, overloaded processes are directly transferred to underloaded ones. This problem is addressed by a number of experts, such as [17,18]. Ref. [19] examined the FC paradigm to optimize task planning with bee swarm algorithms aiming to optimize source usage.

CFC has been investigated as a way to aid in managing SG by many researchers. As a solution, a test case study was discussed for demand response optimization in the SG utilizing the CFC platform. As a result, CC’s performance will be increased in the SG by using FC. Authors in [20] examined the SG information management system using the CC scheme, employing distributed information management for real-time information capturing, ubiquitous accessibility, and real-time data retrieval using parallel processing. In [21], a cloud-driven demand response framework was devised to enable quicker response times in large-scale implementations. There were two kinds of demand response strategies investigated: cloud-driven demand response and dispersed demand response. Moreover, the convergence time was minimized and bandwidth was efficiently utilized. As it can be inferred from these explanations, none of the above works have investigated the role of CFC in SGs including the computational time needed to solve the problem. Therefore, this paper focuses on the use of computational intelligence (CI) methods for improving the efficiency of data transaction systems for multi-objective, multi-level optimization problems in CFC. There are two types of optimization problems, depending on the variables considered: combinatorial problems and continuous problems, respectively, for discrete variables and continuous variables. The paper discusses combinatorial optimization problems in SGs, presenting and analyzing the outcomes. Two novel hybrid algorithms including the gray wolf optimization (GWO) and improved differential evolution (IDE) are proposed which would decrease the computational burden effectively. The study’s main contributions consist of:

- Developing new intelligent optimization algorithms for constructing fast and reliable computational algorithms needed in the CFC architectures including the hybrid versions of the gray wolf optimization (GWO) and improved differential evolution (IDE);
- Proposing a hybrid gray wolf differential evolution (HGWDE) optimization algorithm for SG energy management;
- Comparing the optimization approaches to help the cloud schedules for decreasing the response time and running time of tasks in CFCs.

The remainder of the study consists of below parts: the system scheme, problem definition, and suggested load-balancing method are discussed in Section 2. The suggested optimization approach is presented in Section 3. Section 4 provides a detailed description of the outcomes. The study is summarized and concluded in Section 5.

## 2. System Scheme

**Figure 1:**
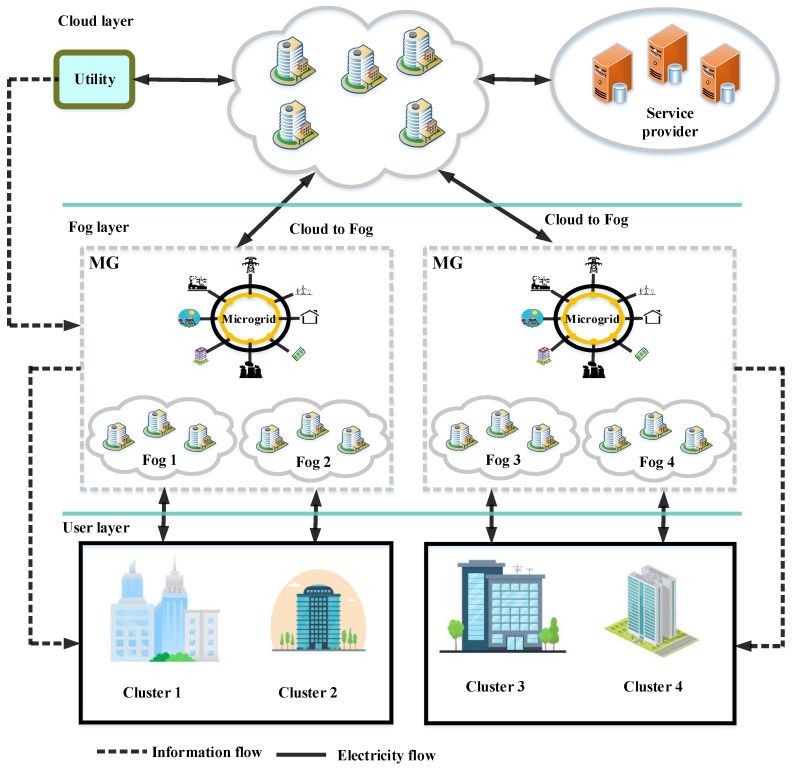
A representation of the suggested system scheme.

There are often several data centers located across the globe for cloud service providers [22]. The described system scheme of the CFC environments consists of several fog data centers and cloud data centers. An example of the SG model is shown in Figure 1, utilizing a geo-distributed CFC environment. Three layers are included in the proposed scheme: the end user, the fog layer, and the core cloud layer. The fog layer consists of a variety of software and hardware components to provide local analyses and monitoring. Users and the cloud are connected via this layer to each other. Moreover, the fog layer is responsible for managing requests from users from various parts of the globe. Data access and energy consumption are included in the requests. Therefore, the cloud load is reduced by the fog. The following part proposes architecture with 3 layers, consisting of a cloud, a user, and a fog. Data is shared between the 3 layers [23]. It is assumed that N buildings with several houses make up the user layer. All residences are equipped with an RER and an energy storage system (ESS) for meeting energy demands [24,25]. It is environmentally friendly and does not emit pollutants due to the fact that the energy is derived from natural resources. Furthermore, the ESS stores surplus produced power at low-production times for meeting domestic load demands [26]. A house’s power consumption, production, and device planning are each received by the fog layer. Cloud services are used by the layer for operating their applications. The fog devices are connected to smart homes or households by smart meters. Each household communicates its energy shortage and surplus data via the CFC environments. The communication among the smart meters is performed through a local area network, a wide area network, or a metropolitan area network.

Cloud extensions such as fog act as a middleman between users and the cloud. All clusters are connected by the fog. Fog layers are used to temporarily store information, immediately prior to moving to the cloud for permanent storage. Fog provides similar characteristics as the cloud but can be more accessible to users. Hence, low-delay services are therefore beneficial to consumers. Virtual machines (VMs) in the fog process requests from customers. A user can request power from microgrids by submitting a request to the fog.

The second layer, called the fog layer, regulates delay and manages network sources effectively. As the fog layer has been located near the clients, it physically takes place at their local place. If the communication and physical distances are similar, the fog node will be near the user.

Another focus of this article is to propose an intelligent and fast cyber-layer for smart grids to record, store, analyze, and manage the big data all around the smart grid. In this way, first, the structure of the cloud-fog-user layer is depicted and described in Figure 1. Second, the appropriate model and algorithms required to control the proposed layer are described. Although these three layers look similar at first glance, they represent different computing resources as different layers of SG. In fact, each layer builds on the capabilities of the previous layer. In Figure 1, the lower level is the user layer, also called the edge layer, and is in charge of edge computing. In other words, this layer will handle some processing of sensor data away from the cloud layer (or central control of the SG). Therefore, the user layer pushes the computations to happen on the edge of the network. The input to this layer can be named as the active and reactive power demand of the system loads, the voltage level of the system buses, the network topology, the renewable energy sources output power, the distributed generators capacity, price and limitations, the market price, etc. It will try not to let data transfer from the end users to the cloud for analysis of action. Therefore, the communication of big data toward the upper layers reduces and thus a simpler chain of communication with less potential for failures would be provided. In the second layer, the fog layer exists which extends cloud computing to a lower layer. This layer tries to push intelligence down to the end users and simulate an analyzing system close to the sensors in SG. Therefore, the fog layer is the first layer in the digital world of the smart grid. On the top of all layers, the cloud layer exists which has the capability of global storage of data, analyzing and launching final commands for the operation of the smart grid. The output of the upper cloud layer would be the power flow outputs including the voltage level of the buses, the power flow in the feeders, the optimal output power of the units, the renewable energy sources, and the operating point of the loads. These fogs have been controlled by internet service providers. To this end, various fogs are present in the fog layer. A fog device has been connected to all smart buildings in this way. A fog device consists of virtualized hardware (H/W). Through virtualization, VM monitors and manages multiple VMs on one computer. Virtual machines (VMs) manage multiple operating systems (OSs) on one H/W platform (VMM). VMMs or hypervisors are interfaces among guest OSs and VMs. VMs, or guest OSs, are central processing units that run multiple programs. User connections to the cloud are made through the fog.

**Figure 2:**
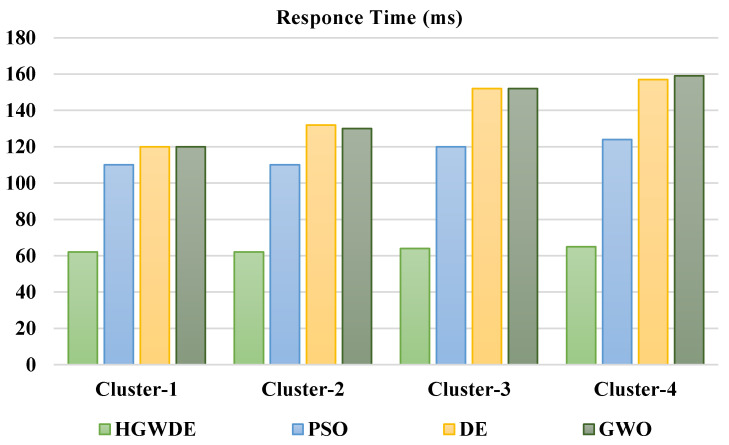
Average response time.

The final layer includes the core cloud. In the cloud layer, information is processed and administered on demand via remote servers. There is an inextricable connection between CFCs. Consumer information is temporarily stored in fogs prior to being sent to the cloud to be permanently stored. A computing application’s computational load profile plays a crucial role in the implementation of CC. Overburdened servers occur whenever there are many applications running on a single platform. According to Figure 2, consumers request numerous visits to the service providers. VMs utilize load balancers to maintain load balance and optimize source usage. Numerous load-balancing methods are used by CC to ensure effective control of computing load profiles. Computing load profiles are the same as power load profiles in SGs. Therefore, if the SGs are integrated with the CFC-driven environments, afterward, tasks associated with the SGs must also be efficiently managed in terms of the computing load profile. The paper uses 4 heuristic solutions to solve the load-balancing issue. In the case of the system scheme being applied to each part of the world, all areas have various numbers of structures and fogs.

As can be seen from the cloud-fog-edge layer, the main purpose is to reduce the communication traffic and push the high computational burden down to the local areas. In this way, each layer should be equipped with appropriate fast and intelligent algorithms. The first task for any optimizer is to have a clear objective function. In Equations (1)–(10), the objective functions including the response time, the latency time, and the price of VMs are modeled. Later, new optimization evolving methods are developed to solve the optimization problem at the appropriate level. Through such an intelligent structure, the power delivery services would enhance greatly in the smart grid.

### 2.1. Problem Definition

There are 3 layers in the offered system framework. A representation of the offered structure is depicted in Figure 1. At the top, the cloud layer stands. The 2nd and sole intermediate layer would be the fog layer. The 3rd and last layers belong to the consumer. Hence, consumers’ requirements are met by the layers communicating among themselves. The suggested structure consists of 6 distinct parts. Users send requests to the fog via the SG for computing and other measuring tasks. As a result of utilizing resources effectively, the fog meets consumer needs. The task group T is expressed as follows:

(1) $$T=T_{1},T_{2},\ldots ,T_{m}$$

Fogs can have the following number of *VM*s:

(2) $$VM=\overset{v}{\underset{i=1}{\sum }}\left(VM_{i}\right)$$

wherein *v* shows the number of *VM*s. The objective function consists of minimizing response time and processing, expressed in the following way:

(3) $$K_{minimizw}=\overset{m}{\underset{j=1}{\sum }}\overset{n}{\underset{i=1}{\sum }}\left(RT*P_{ij}*Delay\right)$$

wherein *RT* shows the response time as in Equation (6) and *Delay* shows the delay time. The fitness function can be determined in the following way:

(4) $$Fitness=Max\left[EXC_{vm_{1}}\left(F_{1}\right),\ldots ,EXC_{vm_{n}}\left(F_{m}\right)\;\right]$$

In which, $EXC_{vm_{1}}\left(F_{1}\right)$ shows the working time for performing the group of tasks on fog $F_{1}$ on $vm_{1}$. $F_{1}$ shows the group of clusters of consumers, meaning, $F_{1}=\left[C_{1},\;C_{2},\;\ldots ,\;C_{x}\right]$, in which x is the count of consumers on fog $F_{1}$. In addition, *n* shows the count of *VM*s and m shows the count of fogs.

#### 2.1.1. Processing Time

*VM* capacity and task length processing time are determined in the following manner:

(5) $$PT=\overset{N}{\underset{i=1}{\sum }}\overset{M}{\underset{j=1}{\sum }}\left(P_{ij}*A_{i}\right)$$

#### 2.1.2. Response Time

The response time has been determined by the difference between the times that the task began to execute and when the customer transmitted the request:

(6) RT=DelayTime+FinishTime−ArrivalTime

#### 2.1.3. Price

The cost of CFC is also an essential parameter. In addition to the *MG* cost, *VM* cost and data transfer (*DT*) cost are also two factors that the cost depends on. The below formulas are used to calculate the total cost.

(7) $$Cost_{Total}=Cost_{DT}+Cost_{VM}+Cost_{MG}$$

As can be seen in Equation (7), the total cost is computed based on the summation prices of *DT*, *VM*, and *MG*, where $Cost_{DT}$ represents the cost of *DT*, $Cost_{VM}$ shows the cost of *VM*, and $Cost_{MG}$ defines the cost of *MG*. *VM* cost and *DT* cost are obtained by Equations (8) and (9), respectively, as follows:

(8) $$Cost_{VM}=\overset{N}{\underset{i=1}{\sum }}\left(VM_{FinalTime}-VM_{InitialTime}\right)*u$$

(9) $$Cost_{DT}=\frac{T_{Total}}{Data_{Used}*\beta }$$

where u shows a fixed factor and β shows a per *GB* transferring price. $VM_{FinalTime}$ and $VM_{InitialTime}$ are the final time and start time of *VM*, respectively. $Data_{Used}$ shows the transmitted data which is used to execute. *VM*’s overall time is shown in Equation (10):

(10) $$Total_{Time}=Finish_{Time}-Start_{Time}$$

where $Total_{Time}$ defines the overall time of *VM*. $Start_{Time}$ and $Finish_{Time}$ are the start time and finish time of *VM* execution.

## 3. Suggested Model

This paper proposes HGWDE as an optimization method that combines two meta-heuristics: IDE and GWO for optimizing energy usage. The three methods have been employed for comparing the efficiency of the suggested model to that of current models.

### 3.1. Improved Differential Evolution (IDE)

IDE shows a new version of DE, offered by Storn in 1995 for the first time. In the algorithm, the primary population has been determined at random. IDE involves four major stages: population production, mutation, crossover, and choice. Equation (11) generates the population at random.

(11) $$X_{i,j}=l_{j}+\left(rand\times \left(u_{j}-l_{j}\right)\right)$$

The goal is to produce a mutant vector by generating three vectors $x_{r1},\;x_{r2},\;x_{r3}$. The target vector belongs to the 1st vector. Equation (12) gives the mutant vector in the following way:

(12) $$V_{i,G+1}=x_{r1,G}+F\left(x_{r2,G}-x_{r3,G}\right)$$

In which, F shows a scaling factor. Following the creation of the mutant vector, 3 test vectors have been produced. Next, the optimal test vector has been chosen for comparing it to the target vector, thereby populating the production with the optimal vector. Equations (13)–(15) can be used to create the first 3 test vectors.

(13) $$u_{j,i,G+1}=\{\begin{array}{c} V_{j,i,G+1}\;\;\;if\;randb\left(j\right)\leq 0.3\\ x_{j,i,G}\;\;\;\;\;\;\;\;\;\;\;\;\;\;\;\;\;\;\;\;\;\;otherwise \end{array}$$

(14) $$u_{j,i,G+1}=\{\begin{array}{c} V_{j,i,G+1}\;\;\;if\;randb\left(j\right)\leq 0.6\\ x_{j,i,G}\;\;\;\;\;\;\;\;\;\;\;\;\;\;\;\;\;\;\;\;\;\;otherwise \end{array}$$

(15) $$u_{j,i,G+1}=\{\begin{array}{c} V_{j,i,G+1}\;\;\;if\;randb\left(j\right)\leq 0.9\\ x_{j,i,G}\;\;\;\;\;\;\;\;\;\;\;\;\;\;\;\;\;\;\;\;\;\;otherwise \end{array}$$

Equations (16) and (17) can be used to create the 4th and 5th test vectors.

(16) $$u_{j,i,G+1}=randb\left(j\right).x_{j,i,G}$$

(17) $$u_{j,i,G+1}=randb\left(j\right).v_{j,i,G}+\left(1-randb\left(j\right)\right).x_{j,i,G}$$

Max. *iter* shows the maximal number of iterations; *POP* shows the whole population, i.e., all potential solutions to home energy management (HEM); and h shows the whole time intervals. CR shows the crossover factor, usually 0.3, 0.6, and 0.9. u, μ, and x show the mutant vector, test vector, and target vector. Table 1 shows IDE with regard to HEM.

### 3.2. Gray Wolf Optimization (GWO)

Wolf hunting behavior and leadership hierarchies are the inspirations for GWO’s meta-heuristic optimization method. Leadership can be divided into 4 levels: alpha α, beta β, delta δ, and gamma γ. As the group’s smartest leader, alpha guides the others on how to hunt. Alpha is followed by delta and beta under the hierarchical structure, and gamma defines at the bottom. Thus, gamma is ineligible for leadership positions. In HEM, alpha would be considered the fittest member to minimize costs. Equation (18) creates a random population at the start:

(18) X(i,j)=rand(POP,D)

In which, *POP* is the entire gray wolf population, and *D* represents all counts of devices. In every search agent, the objective function (the distance from the target) is determined using *A* and *C*.

#### 3.2.1. Encircling Target

Target is encircled by gray wolves prior to the hunt. According to ref. [27], the below formulas can be used to formulate gray wolves’ encircling behavior.

(19) $$X\left(t+1\right)=X_{p}\left(t\right)-A\times D$$

(20) $$D=\left|C\times X_{p}\left(t\right)-X\left(t\right)\right|$$

Here $X_{p}$ shows the location of the target, whereas *X* shows the location of the wolf for the tth iteration, and can be determined via Equation (19). Moreover, Equations (21) and (22) can be used to calculate the vectors *A* and *C*:

(21) $$\vec{A}=2\vec{a}\times \vec{r_{1}}-\vec{a}$$

(22) $$\vec{C}=2\times \vec{r_{2}}$$

In which, $\vec{r_{1}}$ and $\vec{r_{2}}$ show randomly selected vectors in the range [0, 1]. Several iterations reduce a’s value from 2 to 0. *C* varies at random within a range of 0 and 2, indicating the weight for target attractiveness [28].

#### 3.2.2. Hunting

Alpha is the primary guide to the hunt, with beta and delta serving as secondary factors. The alpha leads them because the alpha knows the location of the target most well. Gamma updates its location based on the optimal solution determined by the 1st three members. Equation (23) updates the location of wolves.

(23) $${\vec{X}}_{t+1}=\frac{\vec{x_{1}}+\vec{x_{2}}+\vec{x_{3}}}{3}$$

Equations (24)–(26) can be used to determine $\vec{x_{1}}$, $\vec{x_{2}}$, and $\vec{\;x_{3}}$.

(24) $$\vec{x_{1}}=\vec{x_{\alpha }}-\vec{A_{1}}\times \left(\vec{d_{\alpha }}\right)$$

(25) $$\vec{x_{2}}=\vec{x_{\beta }}-\vec{A_{2}}\times \left(\vec{d_{\beta }}\right)$$

(26) $$\vec{x_{3}}=\vec{x_{\delta }}-\vec{A_{3}}\times \left(\vec{d_{\delta }}\right)$$

Here $\vec{x_{\alpha }}$, $\vec{x_{\beta }}$, and $\vec{x_{\delta }}$ show the optimal solutions achieved in the tth iteration; Equation (21) can be used to determine $\vec{A_{1}}$, $\vec{A_{2}}$, and $\vec{A_{3}}$, and Equations (27)–(29) can be used to determine $\vec{D_{\alpha }}$, $\vec{D_{\beta }}$, and $\vec{D_{\delta }}$:

(27) $$\vec{D_{\alpha }}=\vec{C_{1}}\times \vec{x_{\alpha }}-\vec{x}$$

(28) $$\vec{D_{\beta }}=\vec{C_{2}}\times \vec{x_{\beta }}-\vec{x}$$

(29) $$\vec{D_{\delta }}=\vec{C_{3}}\times \vec{x_{\delta }}-\vec{x}$$

Equation (22) can be used to determine $C_{1}$, $C_{2}$ and $C_{3}$. In the final step, variable a is graded from two to zero in all iterations, controlling the balance between exploitation and exploration based on Equation (30).

(30) $$a=2-t\frac{2}{Max.iter}$$

The objective function is shown in Equation (31) and can be determined by multiplying the power rating by the state of every device.

(31) $$Fitness=\rho_{D}\times Xv_{D}\left(h\right)$$

Max. *iter* shows the maximal iterations, POP shows the entire population, D shows the count of devices, and fitness would be the objective function. α shows the optimal solution or participant in the hunting process, whereas β and δ would be the 2nd and 3rd optimum solutions.

The fitness function has been compared to the fitness of α, β, and δ for evaluating the optimal leader. Equations (27)–(29) can be used to update their last locations.

### 3.3. Hybrid Gray Wolf Differential Evolution (HGWDE)

Please note that the new hybrid algorithm called HGWDE is a direct contribution of this work which is specifically designed for the optimal operation of the SG. The proposed algorithm benefits from the hybridization of the GWO and DE with a new formulation to limit the computational time needed for big data analysis and transactions within the CFC architecture. A detailed discussion of the suggested model has been provided here. IDE generates an updated population through four stages: initialization, mutation, crossover, and choice. In order to update the population, the optimal test vector will be chosen from five vectors and then compared to the target vector. Since IDE takes into account the entire range of test vectors, it is efficient at selecting the optimal trial vector. Encircling targets, hunting, and updating wolf locations make up GWO’s main stages. The leader belongs to  α. Each search agent updates its location based on α. GWO does not compare α to  β or δ. Moreover, β and δ may be nearer to the target than α. Crossover from IDE must be used to compare each search agent. GWO is used to update the location of search agents after the optimal search agent has been selected. The HGWDE was chosen because it combines the most advantageous characteristics of IDE and GWO.

Initialization, encircling the target, selecting the optimal search agent, and updating the location are the stages of HGWDE. To this end, the population of wolves $X_{i}\left(i=1,\;2,\;\ldots ,\;n\right)$ is first generated randomly. Then, three randomly selected vectors are used to create a mutant vector v. α,  β, and δ are initially set up as three vectors. Equation (31) calculates the fitness of v and α,  β, and δ. The crossover will be carried out according to the following formulas:

(32) $$\alpha_{new}=\{\begin{array}{c} v_{j}\;\;if\;fitness\;of\;v_{j}\;\leq \alpha \\ \alpha \;\;\;\;\;\;\;\;\;\;\;\;\;\;\;\;\;\;\;\;\;\;\;Otherwise \end{array}$$

(33) $$\beta_{new}=\{\begin{array}{c} v_{j}\;\;if\;fitness\;of\;v_{j}\;\leq \beta \\ \beta \;\;\;\;\;\;\;\;\;\;\;\;\;\;\;\;\;\;\;\;\;\;\;Otherwise \end{array}$$

(34) $$\delta_{new}=\{\begin{array}{c} v_{j}\;\;if\;fitness\;of\;v_{j}\;\leq \delta \\ \delta \;\;\;\;\;\;\;\;\;\;\;\;\;\;\;\;\;\;\;\;\;\;\;Otherwise \end{array}$$

The location of search agents generally changes following a selection process. Table 2 illustrates the mapping of HGWDE parameters with HEM.

In the beginning, the needed parameters are initialized. Stage 2 generates the population at random. Iterations are adjusted to max once the population has been generated. IDE is used to compare the mutant vector’s fitness to α, β, and δ. GWO updates the location of search agents until the stopping criteria are met.

## 4. Simulation Results and Explanation

This process is likewise implemented as part of Cloud-Analyst, a Cloud-Sim toolkit extension. A graphical user interface is available for Cloud-Analyst as well. In Cloud-Sim, VMs are simulated and deployed with various hardware features. Regarding the simulation results analysis and modeling, the main focus of this article is to assess the role of meta-heuristic algorithms to help the three-layer CFC architecture improve the process of analyzing data, storing data, and managing them in a proper time with the least possible latency. Therefore, the modeling process first comes around the task division among the layers as explained before which happens in the Cloud-Sim toolkit. Any further modeling would come around the optimization algorithms and how they can analyze big data, solve the problem considering the technical limits, and get to the optimal solution with the least computational time and highest accuracy. It is clear that modeling can expand to many more details up to communication channels, the communication traffic layers, etc., which are out of the scope of this paper. A comprehensive analysis and comparison is made among the different algorithms which are explained in the rest of this study.

### 4.1. Response Time Validation

Figure 2 depicts the response time of user clusters with PSO, DE, GWO, and HGWDE algorithms. As can be seen from Figure 2, PSO, DE, and GWO have much longer response times than HGWDE. Optimal response times were achieved for each user cluster at the same time. The fog is reduced by HGWDE’s efficient allocation of resources. Using simulated annealing, HGWDE finds the optimal possible solution for all jobs and plans requests efficiently. As soon as a job arrives, the load balancer determines the VM’s memory, utilization, energy requirement, and speed. In addition, according to the variables, jobs are allocated to the VM with the highest priority, which keeps the procedure moving quickly. The outcomes show that it is considerably better than PSO, DE, and GWO in terms of load balancing. Based on the outcomes, it can be concluded that odds and GWO have similar results. PSO outperformed the odds and GWO [29,30]. Table 3 illustrates the summary of the response times of each method. The HGWDE algorithm outperforms various algorithms.

**Figure 3:**
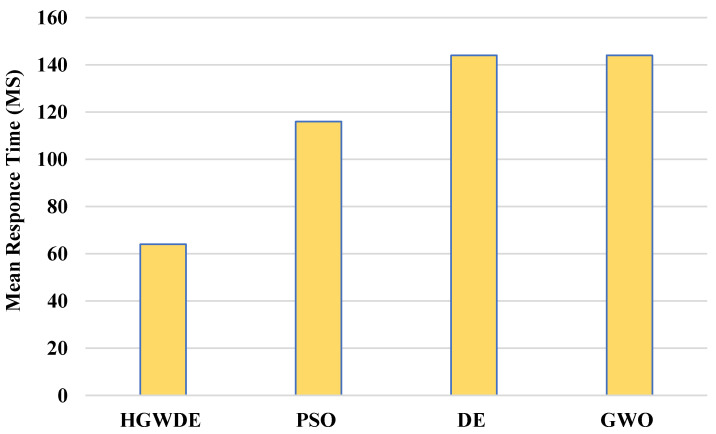
Average response time for clusters.

A PSO reaction takes 116.01 ms, a DE reaction takes 144.10 ms, a GWO reaction takes 143.60 ms, and an HGWDE reaction takes 63.02 ms, according to Figure 3.

### 4.2. Processing Time Validation

**Figure 4:**
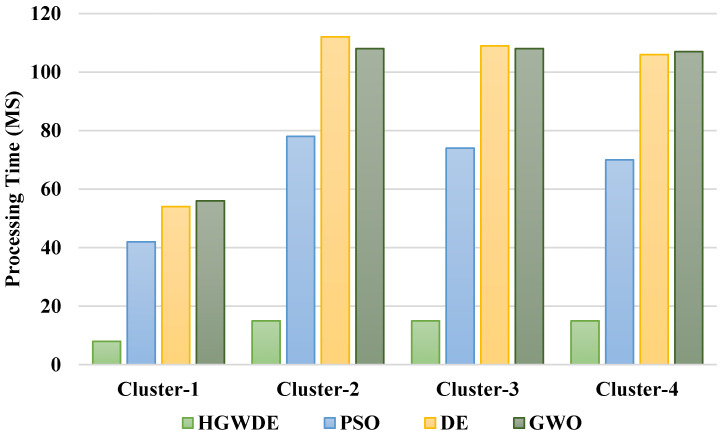
Comparing processing time of various algorithms.

Figure 4 depicts the processing time of user clusters with the PSO, DE, GWO, and HGWDE algorithms. It can be seen from Figure 4 that the processing time for the PSO, DE, and GWO exceeds that for the HGWDE. Optimal processing times were achieved for each user cluster at the same time.

In HGWDE, many agents, known as particles, work together to optimize the fog’s load. A deterministic and stochastic component helps the particles find the optimal solutions. It also performs better because it has fewer coefficients to tune than other optimization techniques. With each iteration, a particle gets nearer to the final ideal solution.

**Figure 5:**
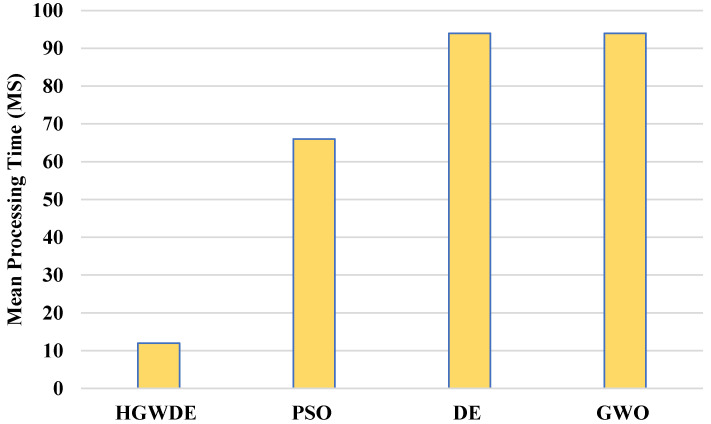
Average processing time of fogs.

According to Figure 5 and Table 4, the average processing time for the PSO is 66.33 ms, for DE is 94.59 ms, for GWO is 93.95 ms, and for HGWDE is 13.39 ms.

### 4.3. Price and Costs Validation

**Figure 6:**
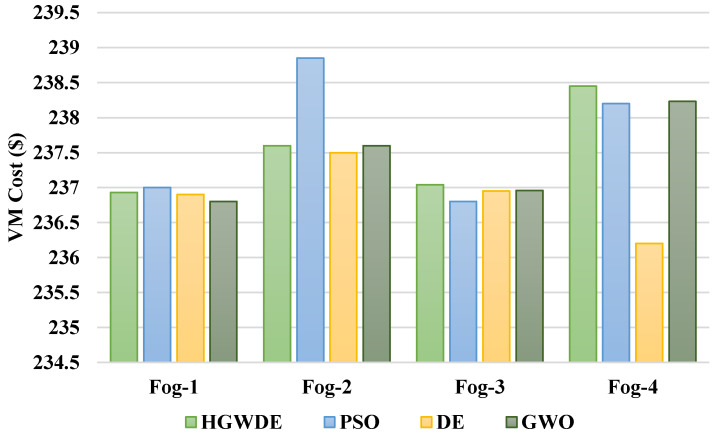
Average VMC for fogs.

**Figure 7:**
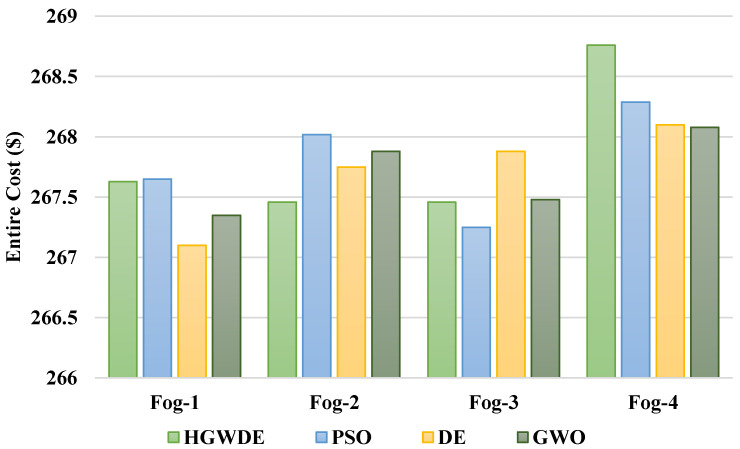
Average overall price for fogs.

The VMC is shown in Figure 6. The VMC for the user clusters with the PSO, DE, GWO, and HGWDE algorithms is shown in Figure 7. Figure 7 shows that the price of the PSO, DE, and HGWDE exceeds that of the GWO. Optimal prices were achieved for each user cluster at the same time.

Based on the outcomes, GWO provides a more cost-effective load-balancing algorithm in comparison to PSO, DE, and HGWDE. GWO and DE methods have similar outcomes, according to the results. With the DE approach, the PSO and GWO performed better. Table 5 provides a summary of the prices associated with the earlier described algorithms.

**Figure 8:**
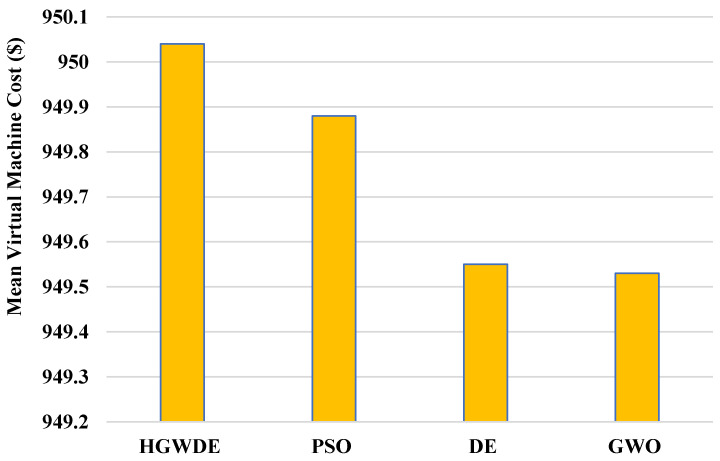
Average VMC.

**Figure 9:**
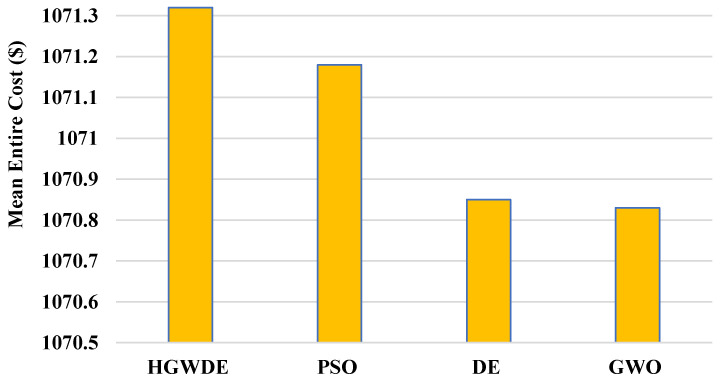
Average price.

According to Figure 8, the average VMC for PSO is 949.9$, for DE is 949.5$, for GWO is 949.5$, and for HGWDE is 950$. According to Figure 9, the average price for PSO is 1071.9$, for DE is 1070.9$, for GWO is 1070.8$, and for HGWDE is 1071.3$.

Simulations indicate that HGWDE performs better than the PSO, GWO, and DE algorithms. As a result of combining PSO’s superior features with simulated annealing, HGWDE exhibits superior efficiency. HGWDE provides the optimal global and GWO provides local solutions. Response time, processing time, running time, and price of the GWO were slightly higher because of its slow convergence. Local optima may prevent GWO from determining the global optimal solution. As a way to meet GWO limitations in the future, ABC fitness could be added to the algorithm, resulting in faster reaction times, processing times, costs, and execution times as a result of its higher convergence rate.

## 5. Conclusions

This paper presents an intelligent and optimal structure for a CFC-driven environment integrated with SG. There are three layers in the scheme: a cloud, a fog, and a consumer. The cloud layer consists of cloud servers, the fog layer consists of fog servers and VMs, and the end-user layer contains buildings, which will cover a number of home consumptions. A novel scheme using improved DE and GWO algorithms, called HGWDE, was proposed to reduce the latency and enhance the model efficiency. In HGWDE, the inertia weight is used to regulate search space sizing so that the optimal solution can be found. A comparison is made among HGWDE, PSO, DE, and GWO. There is a slight advantage for GWO in terms of price performance, but the difference is not significant. The simulation results clearly show the high performance and capabilities of the proposed model for managing smart grids. Moreover, it was seen that the successful performance of an SG relies on big data transaction management and analysis which is made possible only through a fast, precise, and reliable architecture. The proposed CFC is a necessity for the optimal and successful performance of the SG considering the big data transactions happening within the system. The simulation results clearly advocate the necessity of smart and intelligent optimization algorithms embedded within the CFC, which is proposed by HGWDE in this paper. Not only the accuracy of the proposed hybrid algorithm is validated through several comparisons made for the optimal scheduling of the SG, but also very little computational effort is needed to show its fitting role for CFC architecture. The authors would address the effect of the proposed cyber-layer based on metrics and measures in future works.

## Figures and Tables

**Table 1:** IDE mapping on HEM.

**IDE variables**	Population	Number of dimensions	Gradient of problem
**HEM variables**	Feasible solution	Number of appliances	Scheduling
**Amounts**	50	17	Vary

**Table 2:** HGWDE mapping on HEM.

Variable	HEM			
	Feasible Solution	Devices	Minimum (Price)	State of the Devices
**Amount**	50	17	vary	1 or 0
**Variable**	**HGWDE**			
	**Population**	**Wolfs in all packs**	**Minimal distance form target**	**State of the leader**
**Amount**	50	17	vary	1 or 0

**Table 3:** Overview of response time for each algorithm.

Algorithms	HGWDE	PSO	DE	GWO
**Max. (ms)**	87.3	584.7	581.4	577.1
**Min. (ms)**	38.7	41.8	42.6	42.6
**Average (ms)**	63	116	144.1	143.6

**Table 4:** Overview of processing time for each algorithm.

Algorithms	HGWDE	PSO	DE	GWO
Max. (ms)	26.1	531.6	530.5	530.3
Min. (ms)	0.2	0.3	0.6	1.1
Average (ms)	13.4	66.3	94.6	93.9

**Table 5:** Overview of price for each algorithm.

Algorithms	HGWDE	PSO	DE	GWO
Overall price (USD)	1071.3	1071.2	1070.9	1070.8
Information transfer price (USD)	121.3	121.3	121.3	121.3
VM price (USD)	950	949.9	949.6	949.5

## Data Availability

Not applicable.

## Nomenclature

T	Task group	$X_{i,j}$	Population
m	Count of fogs	$X_{p}$	Location of the target
v	Number of virtual machines	$\vec{r_{1}}$ and $\vec{r_{2}}$	Randomly selected vectors between [0, 1]
n	Count of VMs	α	Best search agent
m	Count of fogs	β	2nd best optimal solution
x	Count of consumers on fog $F_{1}$	δ	3rd best optimal solution
u	Fixed factor	$\vec{x_{\alpha }}$, $\vec{x_{\beta }}$ and $\vec{x_{\delta }}$	Optimal solutions achieved in the $t^{\mathrm{th}}$ iteration
β	Per GB transferring price	$D_{\alpha }$	Position of the α search agent
$P_{\mathrm{ij}}$	Length of task	$D_{\beta }$	Position of the β search agent
$A_{i}$	Task specified for every request	$D_{\delta }$	Position of the δ search agent
${\mathrm{Arrival}}_{\mathrm{Time}}$	The time when the task began	F	Scaling factor
${\mathrm{Finish}}_{\mathrm{Time}}$	The time when the task execution is finished	v	Mutant vectors
${\mathrm{Delay}}_{\mathrm{Time}}$	Delay time from customer transmitting the request till the task began to execute	Max. iter	Maximal number of iterations
${\mathrm{Cos}t}_{\mathrm{DT}}$	Cost of DT	POP	Whole population
${\mathrm{Cos}t}_{\mathrm{VM}}$	Cost of VM	h	Whole time intervals
${\mathrm{Cos}t}_{\mathrm{MG}}$	Cost of MG	CR	Crossover factor
${\mathrm{VM}}_{\mathrm{final}\;\mathrm{time}}$	The final time of VM	u	Mutant vector
${\mathrm{VM}}_{\mathrm{InitialTime}}$	Start time of VM	μ	Test vector
${\mathrm{Data}}_{\mathrm{Used}}$	Transmitted data which is used to execute	x	Target vector
${\mathrm{Total}}_{\mathrm{Time}}$	The overall time of VM	D	All counts of devices
$P_{\mathrm{ij}}$	Length of task	A and C	Fitness coefficients
$K_{\mathrm{minimizw}}$	Minimizing response time and processing	X	Location of the wolf for the $t^{\mathrm{th}}$ iteration
${\mathrm{EXC}}_{{\mathrm{vm}}_{1}}\left(F_{1}\right)$	Working time of performing the group of tasks on fog $F_{1}$ on ${\mathrm{VM}}_{1}$	a	Graded from two to zero in all iterations
$F_{1}$	Group of clusters of consumers	PT	Processing time
${\mathrm{Start}}_{\mathrm{Time}}$	The start time of VM execution	RT	Time duration from when a request of uses is sent to fog and when a response has been obtained
${\mathrm{Finish}}_{\mathrm{Time}}$	The finish time of VM execution	VM	Virtual machines
$l_{j}$ and $u_{j}$	Lower and upper bounds		
